# Patient-Derived Meningioma Organoids: A Reliable Model for Studying Human Tumor Pathophysiology

**DOI:** 10.3390/cancers17030526

**Published:** 2025-02-05

**Authors:** Youssef M. Zohdy, Arman Jahangiri, Fadi Jacob, Aliaksandr Aksionau, Ali M. Alawieh, Amelia Tong, Bethany Chern, Justin Maldonado, Kimberly Hoang, Edjah Nduom, Brian M. Howard, Daniel L. Barrow, Stewart G. Neill, Zhexing Wen, Gustavo Pradilla, Tomas Garzon-Muvdi

**Affiliations:** 1 Department of Neurosurgery, School of Medicine, Emory University, Atlanta, GA 30322, USA; youssef.ismail@emory.edu (Y.M.Z.);; 2 Department of Pathology, Emory University, Atlanta, GA 30322, USA; 3 Department of Cell Biology, Emory University, Atlanta, GA 30322, USA

**Keywords:** organoids, meningioma

## Abstract

***Introduction:*** Meningiomas are the most common primary central nervous system tumors, constituting 39.7% of intracranial tumors. Although generally benign, some exhibit aggressive behavior and risk of recurrence, necessitating adjuvant therapy and repeat surgical interventions. Molecular studies have identified tumor-driving mutations, leading to targeted therapies and clinical trials. However, translating preclinical findings into clinical success is often hindered by limitations in current meningioma tumor models. This study aims to develop and validate a standardized protocol for establishing patient-derived meningioma organoids (MEN-Os) that faithfully replicate human disease. ***Methods:*** MEN-Os were successfully established from 15 meningioma samples (11 grade 1, 4 grade 2) from neurosurgical resections using an optimized culture protocol. Histological and immunohistochemical analyses were used to assess the resemblance of MEN-Os to original tumor tissues. RNA sequencing compared transcriptional signatures between MEN-Os and corresponding patient-resected tissues. ***Results:*** MEN-Os were successfully established from patient-resected samples and maintained in culture for up to four weeks, showing stable growth and structural integrity. Histopathological analysis revealed that MEN-Os preserved key architectural features, including cellular organization, nuclear morphology, and proliferation rates. Immunohistochemical staining for meningioma-specific markers, such as the progesterone receptor, confirmed similar expression patterns to parental tumors. Transcriptomic profiling demonstrated that MEN-Os retained the transcriptional signatures of original tissues, including genes associated with meningioma pathology (NF2, CDKN2A, TP53). Differential expression and deconvolution analyses showed that MEN-Os contained diverse cell populations, including tumor and stromal cells, while preserving the immune microenvironment, as validated by histopathological and transcriptomic profiling. ***Conclusion:*** We established a robust, reproducible protocol for generating MEN-Os, which faithfully replicates the histopathological, molecular, and cellular characteristics of original tumors. MEN-Os provide a valuable model for studying meningioma biology and evaluating therapeutic strategies.

## Simple Summary

Meningiomas are the most common brain tumors and are usually benign, but some tend to be invasive, grow back after treatment, and require additional surgeries or therapies. Current tumor models do not fully mimic human meningiomas, making it difficult to study the disease and develop new treatments effectively. To address this gap, we developed a method to grow three-dimensional models of meningiomas, called organoids, directly from patient tumor samples. These organoids resemble the original tumors microscopically and share similar genetic and molecular features. They contain diverse cell types, including stromal and immune cells, which are important for studying how tumors interact with their environment. This research provides a reliable tool for better understanding meningiomas and testing potential therapies, with the goal of improving treatment strategies for patients.

## 1. Introduction

Meningiomas are the most prevalent primary tumors of the central nervous system (CNS), representing 39.7% of all intracranial tumors [1]. Due to their typically benign nature, these tumors are often managed conservatively, with surgical intervention reserved for symptomatic or growing tumors. Meningiomas are classified according to the World Health Organization (WHO) grading system, which categorizes them into three grades based on histopathological and recently, molecular features [2,3]. Grade 1 tumors, which are benign, are associated with a favorable prognosis and a low likelihood of recurrence. Grade 2 and grade 3 meningiomas exhibit more aggressive, invasive behavior and carry a higher risk of recurrence, often necessitating postoperative adjuvant therapy [4].

Large, multicenter cohort studies have demonstrated that a subset of grade 1 meningiomas can recur or exhibit more aggressive, higher-grade-like behavior despite their benign histopathological classification [4,5,6]. Conversely, some patients with grade 2 or 3 meningiomas may experience prolonged postoperative stability [4,5,6]. An additional challenge in managing these tumors is the development of effective adjuvant therapies. The molecular characterization of meningiomas has led to the identification of key tumor-driving mutations, including NF2, POLR2A, TRAF7, KLF4, AKT1, SMARCB1, SMARCE1, and SMO [7,8]. These discoveries have led to the development of targeted molecular therapies and clinical trials [9]. Nevertheless, preclinical successes often fail to translate into meaningful clinical outcomes [10]. A major contributing factor to this gap is the limitation of current in vitro and in vivo models, which struggle to faithfully recapitulate the complexity of human meningioma biology, hindering the effectiveness of therapeutic strategies.

Meningiomas are slow-growing tumors. This complicates the establishment of traditional 2D cultures, making meningioma research more challenging compared to other CNS tumors. These tumors often require additional genetic and phenotypic alterations to sustain long-term cultures, which can compromise the fidelity of human tumor cells in vitro [11,12]. Xenograft models also face limitations, particularly due to the immunodeficient nature of the commonly used models [13], which fail to capture the critical role of the tumor’s immune environment—an essential contributor to meningioma pathology [14]. In response to these limitations, tumor organoid culture models have gained significant attention in the past decade as a more faithful alternative. Initially developed to mimic the physiology of healthy human organs, including the brain and gastrointestinal tract, organoid models have gained increasing interest for their ability to replicate human cancers, including liver, lung, and, notably, glioblastomas [15,16,17]. Tumor organoids offer several advantages, including the ability to more accurately reproduce the three-dimensional architecture, heterogeneity, and tumor microenvironment found in human cancers [17,18]. Patient-derived meningioma organoids have been previously developed, but current protocols face several limitations. These include an inability to preserve the native tumor microenvironment, reliance on exogenous growth factors, serum, and artificial extracellular matrices, as well as insufficient sample sizes [19,20,21,22]. These shortcomings hinder the accurate establishment of the tumor’s biological complexity, impacting the model’s utility for research and therapeutic testing.

To address these challenges, in this study we present a standardized, reproducible protocol for establishing meningioma organoids (MEN-Os) from patient-resected tumor tissues. The organoids were confirmed to closely resemble the original tumor tissues through histopathological analysis, assessing tumor architecture, proliferative rates, and cellular characteristics. The preservation of the tumor immune microenvironment was also investigated. Additionally, transcriptomic analysis was conducted, demonstrating the ability of MEN-Os to recapitulate the molecular features of the original meningiomas. Our findings highlight the potential of MEN-Os as a reliable and biologically relevant model for studying human disease, offering a valuable platform for studying meningioma pathophysiology and tumor therapeutics.

## 2. Methods

### 2.1. Patient Cohort and Specimens

Tissue samples were collected from patients undergoing meningioma tumor resection at Emory University Hospitals. All samples were surplus to those needed for diagnostic purposes and were specifically gathered for research. The study was approved by the Institutional Review Board, and informed consent was obtained from all patients for the use of their tumor tissues. Prior to surgery, each patient underwent preoperative magnetic resonance imaging, which facilitated intraoperative navigation and assisted surgeons during the tumor removal and tissue collection process. The resected tumors were then analyzed histopathologically and graded according to WHO classification by an independent neuropathologist [2]. In total, 19 meningiomas from various anatomical sites were collected during the study period (Table 1). Given the pilot nature of this study protocol, no formal sample size calculation was performed. Resected tumor tissue was placed in chilled sterile Dulbecco’s phosphate buffered saline with calcium and magnesium (DPBS++) on ice, and immediately transported to the laboratory for processing.

### 2.2. Tissue Processing

Upon receiving the tumor tissue at the laboratory, different samples of the tumor were flash frozen, sectioned using a cryostat on slides, and stained using hematoxylin and eosin, to ensure the use of high-quality tumor cell density tissues for organoid establishment and further downstream analysis (Supplementary Figure S1). Pieces of optimal tissue specimens were placed in chilled Hibernate A medium (Thermo Fisher Scientific, Waltham, MA, USA) supplemented with 1X GlutaMax (Thermo Fisher Scientific) and 1X Antibiotic-Antimycotic (Thermo Fisher Scientific) and transferred to a sterile dish for dissection within a biosafety cabinet. Necrotic tissues were dissected, and the remaining tumor tissue was minced into approximately 0.5–1 mm diameter pieces using fine scissors and then washed with DPBS++. The tissues were then incubated in 1X Lysis buffer (Thermo Fisher Scientific) for 10 min at room temperature under gentle rotation to lyse contaminating red blood cells and then washed again with DPBS++. A few tumor pieces were sampled randomly, and flash frozen for bulk RNA sequencing. For histological analysis, tumor pieces were fixed in 4% paraformaldehyde overnight in 4 °C and then either dehydrated and imbedded paraffin, or incubated overnight at 4 °C in 30% sucrose for cryoprotection; the latter were then flash frozen in optimal cutting temperature compound (OCT) in plastic cryomolds. Frozen tissues were stored at −80 °C until further processing.

### 2.3. Meningioma Organoids Establishment

The organoid establishment protocol was adapted from previously published work on the successful establishment of glioblastoma organoids [17]. The processed tumor pieces were washed with DMEM/F-12 (Thermo Fisher Scientific) and distributed in untreated 6-well culture plates with 4 mL organoid medium: 50% DMEM/F-12 (Thermo Fisher Scientific), 50% Neurobasal (Thermo Fisher Scientific), 1X GlutaMax (Thermo Fisher Scientific), 1X MEM Non-Essential Amino Acids (Thermo Fisher Scientific), 1X Penicillin-Streptomycin (Thermo Fisher Scientific), 1X N2 supplement (Thermo Fisher Scientific), and 1X B27 without vitamin A supplement (Thermo Fisher Scientific). The plates were then incubated in a 37 °C, 5% CO_2_, and 90% humidity sterile incubator on an orbital shaker set at 120 RPM. Medium used for the first 48 h was supplemented with a 1:1000 ROCK inhibitor (Y-27632; StemCell Technologies, Vancouver, BC, Canada) to decrease cellular apoptosis. Culture medium was changed thereafter every 2–3 days. MEN-Os were sampled for histological analysis and RNA sequencing using the same protocols used for the corresponding parent tumor pieces. Importantly, multiple MEN-Os (*N* = 10–15) were collected at each timepoint to ensure a representative sampling of the tumor’s heterogeneity.

### 2.4. MEN-O Growth Analysis

Images were captured using a brightfield microscope at 0, 2, and 4 weeks. Changes in size were quantified by measuring the 2D projected area of tissues/organoids, outlined manually for accuracy. Tissue circularity was assessed using the *Circularity* plugin, where a value of 1 represents a perfect circle. All analyses were performed using ImageJ software 2.14.

### 2.5. Histological Analysis

For H&E and immunohistochemistry (IHC) staining of paraffin-embedded tissues, tissues were sectioned using a microtome and stained using standard protocols by the Histology and Molecular Pathology Core, Emory National Primate Research Center, Emory University.

### 2.6. Immunofluorescence Staining

For immunofluorescent staining, fixed tumor tissues and MEN-Os embedded in OCT were sectioned using a cryostat into 25 µm sections and placed on charged glass slides. Sections were outlined with a hydrophobic pen (Vector Laboratories, Newark, CA, USA) and washed 3 times with TBS with Tween (TBST) (Thermo Fisher Scientific) and then blocked and permeabilized using a solution containing 0.5% Triton X-100, 0.1% gelatin, 1% BSA, 10% donkey serum, and 22.52 mg/mL glycine in TBST for 1 h at room temperature. Tissues were then incubated with primary antibodies overnight in 4 °C (Supplementary Table S1). Tissues were washed 3 times with TBST and incubated in secondary antibodies plus DAPI for 1.5 h at room temperature, then washed with TBST followed by mounting, cover-slipping, and sealing with clear nail-polish.

### 2.7. RNA Extraction, Purification and Sequencing

The frozen samples were thawed, and RNA extraction was promptly initiated. To ensure uniformity, all samples were processed at the same time to minimize potential batch effects. RNA extraction and purification were carried out using the Direct-zol RNA Microprep Kit (Cat. #R2063; Zymo Research, Irvine, CA, USA) following the manufacturer’s guidelines. Nuclease-free water was used throughout the RNA isolation process. The quality and concentration of the extracted RNA were immediately assessed using Nanodrop UV spectrophotometry. After assessment, the RNA samples were stored at −80 °C until ready for sequencing. RNA-seq libraries were prepared using the Illumina mRNA sample prep kit (Cat. #RS-100-0801; Illumina, San Diego, CA, USA) following the manufacturer’s protocol, with three technical replicates per sample. The size and concentration of the library constructs were confirmed using a bioanalyzer before sequencing on the Illumina NovaSeq6000 platform. The quality of the raw sequencing reads was evaluated to ensure successful library preparation and sequencing.

### 2.8. RNA Sequencing Data Analysis

Raw RNA reads were processed using the validated nf-core bulk RNA-seq bioinformatics pipeline [23]. *FastQC* (V 0.12) [24] was used to assess adapter sequences and low-quality reads, which were subsequently trimmed with *TrimGalore* (V 0.6.10) [25]. The trimmed RNA-seq reads were then aligned to the human reference genome hg38 using *STAR* v2.7.11 [26] and quantified with Salmon [27]. Differential gene expression analysis was performed using *DESeq2* (V 1.12) [28] in R (R Core Team, 2022). Principal component analysis (PCA) was performed using the *PCAtools* (V 2.1) [29] package in R.

### 2.9. Bulk RNA Deconvolution

For bulk RNA deconvolution, gene expression profiles were normalized using transcripts per million (TPM). The CIBERSORTx algorithm was employed, leveraging cell type-specific gene expression signatures to infer the relative composition of mixed cell populations. A single-cell RNA sequencing reference was constructed by merging data from six previously sequenced meningiomas (GSE: GSE183655) [30]. Single-cell sequencing cells were downloaded and analyzed using the *Seurat* (V4) package [31] using default parameters. Cell populations were then annotated using the *SingleCellExperiment* (V1.28) package [32]. This reference matrix was then used as input for the deconvolution and compared against the counts matrix from the bulk RNA sequencing data.

### 2.10. Transcriptomic Mutations Analysis

To analyze transcriptomic mutations, we utilized the rnaseqmut pipeline (https://github.com/davidliwei/rnaseqmut, accessed on 20 January 2025). Aligned and sorted reads were processed through the pipeline alongside the reference genome, enabling mutation calling, which identify single nucleotide polymorphisms (SNPs) and insertions/deletions (indels) directly from RNA-seq data. The results were compared between the original tumor tissues and their corresponding MEN-Os. Mutation similarity was analyzed at the SNP/indel level by assessing the concordance of mutant transcript fractions.

### 2.11. Statistical Analysis

Data analysis was performed using GraphPad Prism v.9.3 (GraphPad, San Diego, CA, USA). Quantitative data were expressed as mean ± standard error of mean (SEM). Analysis between measurement was compared using Student’s *t*-test. Sample correlation was investigated using Pearson’s analysis. Variations in the data were considered statistically significant at a *p* value < 0.05.

## 3. Results

### 3.1. Establishing Meningioma Organoids

**Figure 1:**
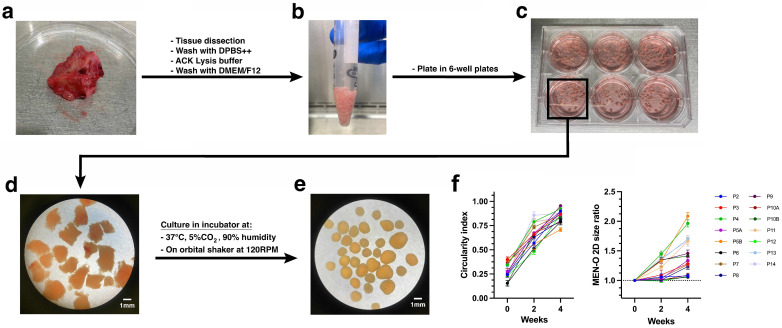
Establishment of patient-derived meningioma organoids. (**a**) Meningioma tissues were obtained from patients undergoing neurosurgical tumor resection. (**b**) Tumor tissues were minced into 0.5–1 mm diameter pieces and washed with DPBS++, lysis buffer, then DMEM/F12. (**c**) Processed tumor pieces were then plated in untreated 6-well plates and cultured at 37 °C, 5% CO_2_, and 90% humidity on an orbital shaker for up to 4 weeks. Bright-light pictures showing tumor pieces at day 0 (**d**) and 4 weeks in culture (**e**). (**f**) Dot plots showing the change in organoid circularity (**left-panel**) and 2D size normalized to timepoint zero (**right-panel**). Dots and error bars represent mean ± SEM. DPBS++, Dulbecco’s phosphate buffered saline with calcium and magnesium; RPM, rotations per minute. CO_2_.

MEN-O culture success was defined by the formation of spherical structures, sustained growth in culture for at least two weeks, and confirmation of patient-tumor-like features through histopathological analysis. MEN-Os were successfully established in 79% of samples (*N* = 15/19). To maintain the original tumor’s cytoarchitecture and preserve cellular populations, we adopted an established protocol for generating meningioma organoids (MEN-Os) which does not subject the resected tumor tissue to mechanical or enzymatic dissociation into single cells (Figure 1). Additionally, the medium used for establishing and sustaining MEN-Os is serum-free and lacks growth factors or extracellular matrix components that could otherwise promote selective growth of certain cell populations. Upon receiving the surgically resected meningioma tumor tissue from patients, an initial preprocessing step was conducted to ensure the establishment of high-quality MEN-Os. The tumor tissue was randomly sampled followed by flash freezing, H&E staining and histopathological examination. Tissues with a high density of tumor cells were selected for establishing MEN-Os, in contrast to the tumor pieces with a high degree of fibers tissue which led to culture failure (Supplementary Figure S1). Of note, multiple tumor tissue sampling is essential to ensure the establishment of a representative MEN-O culture, ensuring the inclusion of tumor heterogeneity. The cell-dense tumor tissues were cut into 0.5–1 mm diameter pieces using fine scissors (Figure 1a,b) and incubated on an orbital shaker to enhance tissue oxygenation and nutrient diffusion. Tumor fragments typically developed into spherical organoids within 2 weeks (Figure 1e,f). Notably, there was a minimal increase in the overall size of the MEN-Os, which is consistent with the slow growth rate of human meningiomas (Figure 1f).

### 3.2. Histopathological Analysis of MEN-Os

**Figure 2:**
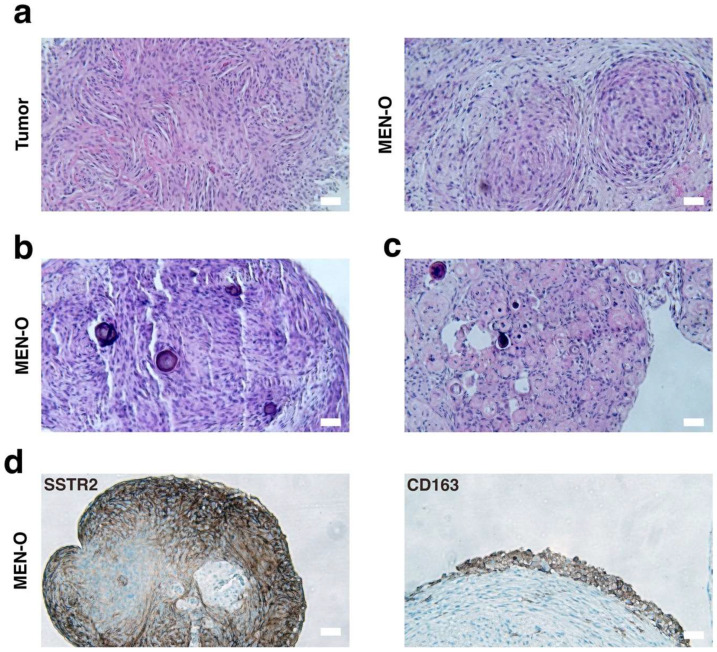
Histopathological analysis of patient-resected meningioma and corresponding meningioma organoid. (**a**) H&E staining of a patient resected meningioma (**left panel**) and corresponding established organoid (**right panel**) showing recapitulation of the tumor’s histological cytoarchitecture and cellular composition. (**b**) Organoid containing psammoma bodies, likely retained from the original tumor given the short incubation period. (**c**) Organoid displaying characteristic whorl pattern seen in meningiomas. (**d**) Strong SSTR2 immunostaining in tumor organoids (**left panel**) and CD163 immunostaining of tumor organoids highlighting tissue macrophages/histiocytes (**right panel**). MEN-O, meningioma organoid; SSTR2, somatostatin receptor 2. Scale bar, 100 µm.

To evaluate whether the MEN-Os resembled their corresponding parental tumors, we first conducted histological analyses. Both the parent tumors and their matched MEN-O samples were examined by a neuropathologist. The MEN-Os faithfully recapitulated the tumor’s cytoarchitecture and cellular composition (Figure 2a). Similar to human meningiomas, the MEN-Os displayed characteristic whorl patterns (Figure 2c), where tumor cells are arranged in concentric circles—a hallmark feature of meningioma histopathology. The organoids also contained psammoma bodies, likely retained from the original tumor rather than newly formed in vitro, given the short incubation period (Figure 2b).

**Figure 3:**
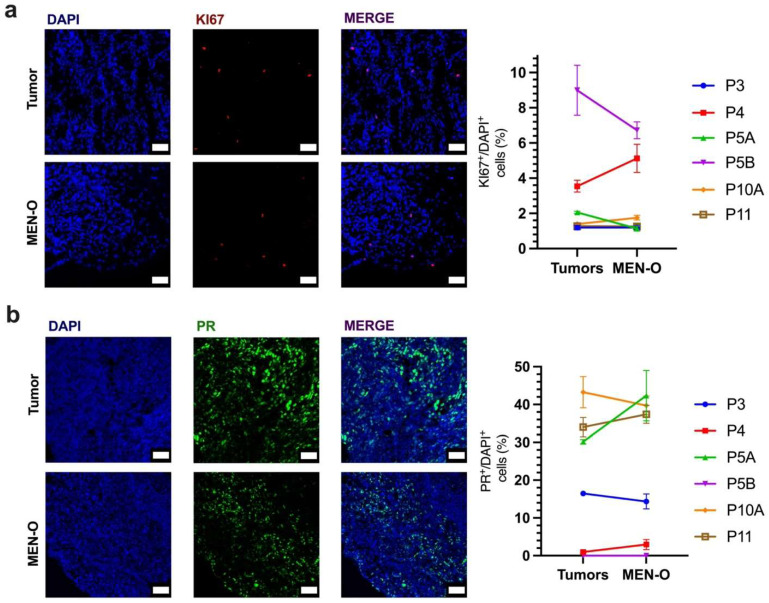
Immunofluorescent analysis of patient-resected meningioma and corresponding meningioma organoid. (**a**) KI67 staining of original tumor tissues and corresponding tumor organoids to analyze and quantify cellular proliferation. (**b**) Progesterone receptor (PR) staining and quantification of the patient-resected meningioma and corresponding organoids. Scale bar, 50 µm. Error bars represent ±SEM.

To further characterize the cellular population in the MEN-Os, we performed immunohistochemical (IHC) analyses using a panel of different markers (Supplementary Table S1). Somatostatin Receptor 2 (SSTR2) is typically overexpressed in meningiomas and serves as a marker for meningothelial cells. IHC staining revealed strong SSTR2 positivity in the MEN-Os, confirming their resemblance to human meningioma tumors (Figure 2d, left-panel). Additionally, the MEN-Os displayed heterogeneous SSTR2 expression across the organoid, reflecting the cellular diversity within the organoid landscape. Progesterone receptor (PR) expression is another characteristic marker of meningiomas, with 70–80% of tumors exhibiting some degree of PR positivity. The percentage of PR-positive cells varies across tumors and has been shown to correlate with tumor grade and prognosis. IHC staining for PR in primary tumors and their corresponding MEN-Os revealed similar PR positivity, with insignificant differences in the number of PR-positive cells (Figure 3b).

We also investigated the proliferation of the MEN-O cellular composition. KI67 immunostaining revealed that the proliferative rate was largely maintained between the parent tissues and the corresponding MEN-Os, indicating comparable levels of cell proliferation in the organoid model (Figure 3a).

Finally, histopathological examination revealed that the MEN-Os not only preserved the tumoral cellular population but also maintained key immune cells within the tumor microenvironment. IHC staining of the MEN-Os confirmed the presence of immune cells, including macrophages (Figure 2d, right-panel).

### 3.3. Transcriptomic Analysis of MEN-Os

**Figure 4:**
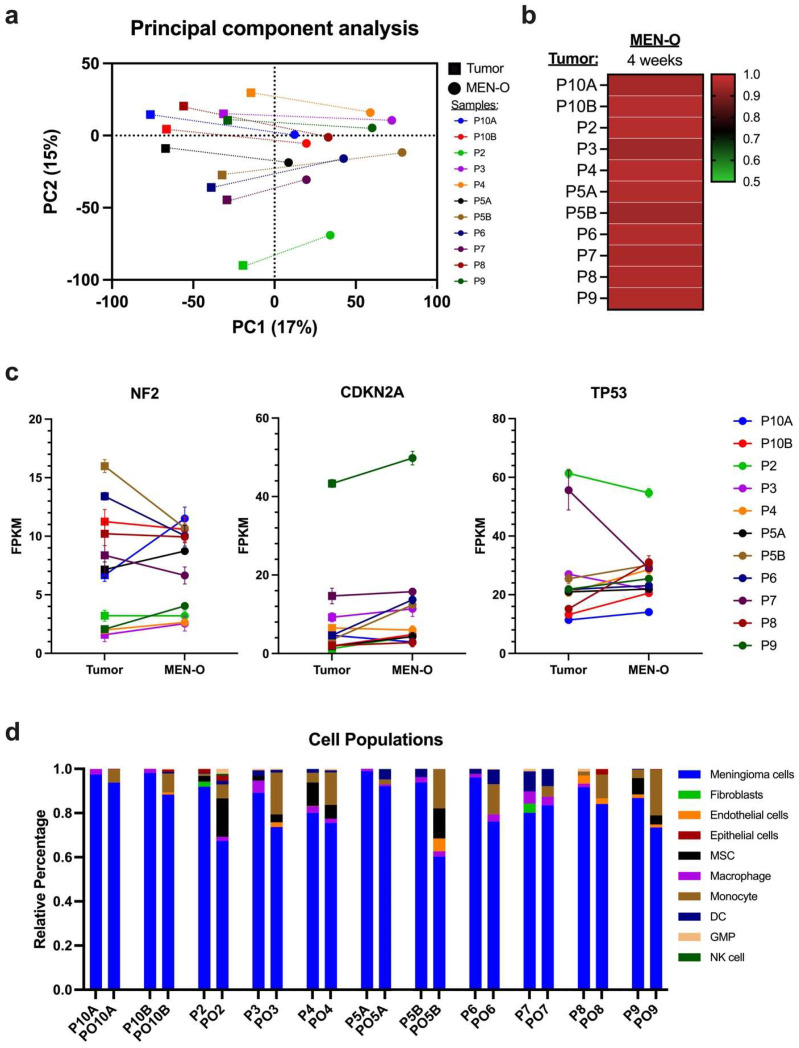
Transcriptomic analysis of patient-resected meningioma and corresponding meningioma organoid. (**a**) Principal component analysis of the original tumor and corresponding established organoids at 4 weeks showing a degree of right cultural shift on PC1, yet little-to-no variation on PC2. (**b**) Heatmap showing Pearson’s correlation (r) between original tumor and corresponding organoids (*p* < 0.001). (**c**) Expression analysis of specific genes in original tumors and corresponding organoids at 4 weeks. Dots and error bars represent mean ± SEM. (**d**) Deconvolution analysis showing the percentage of cellular composition of the original tumors and tumor organoids. MEN-O, meningioma organoids; MSC, mesenchymal stem cells; DC, dendritic cells; GMP, granulocyte/macrophage progenitors; NK cell, natural-killer cell. P#, patient sample (original tumor); PO#, patient organoid (MEN-O).

We investigated whether MEN-Os preserved gene expression profiles from their corresponding parent tumors. Bulk RNA sequencing was performed on parent tumors and respective MEN-Os after 4 weeks in culture. Dimensional reduction of the overall gene expression signatures revealed low PC1 and PC2 variability, a degree of right cultural shift on PC1, yet little-to-no variation on PC2 and a relative proximity between the parent tumors and their corresponding MEN-Os (Figure 4a). Transcriptome-wide analysis showed a high degree of similarity between the MEN-Os and their parent tumors (Figure 4b and Figure S2). Recognizing the inter-tumoral heterogeneity in meningiomas, we analyzed the gene expression profiles of individual parent tumors and observed that their respective MEN-O models preserved these unique expression patterns during culture (Figure 4c). For example, meningiomas exhibited two distinct clusters based on NF2 expression, and the corresponding MEN-Os maintained these differential expression levels. Overall, these findings demonstrate that MEN-Os largely preserve the transcriptomic signatures of their parent tumors, reflecting both inter- and intra-tumoral landscapes.

To analyze the cellular composition of the meningiomas and MEN-Os, the bulk RNA sequencing data was deconvoluted. Single-cell RNA sequencing data from six meningiomas were obtained [30]. Cell clusters were then identified using dimensionality reduction analysis, and cell populations with similar expression signatures were concatenated. A final reference matrix was then constructed (Supplementary Figure S3). Deconvolution analysis revealed an overall retention of different tumoral cellular populations in the MEN-Os. Meningioma tumor cells and stem cells were retained at varying proportions, closely reflecting the parent tumor population (Figure 4d). Additionally, non-neoplastic cell populations, including endothelial cells and immune cells such as macrophages and dendritic cells, were also preserved in the MEN-Os.

**Figure 5:**
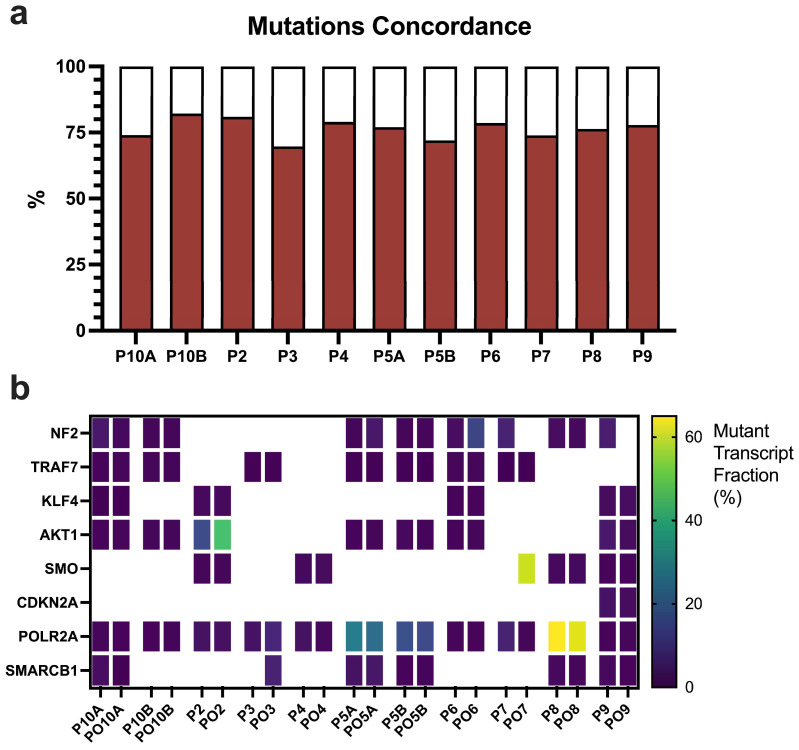
Transcriptomic mutation analysis of original tumors and MEN-Os. (**a**) Percentage overlap of SNPs and indels between original tumors and their corresponding MEN-Os. Calculated as nonsignificant difference in mutation frequency using 2-tailed *t*-test at *p* < 0.05. (**b**) Mutant transcript fractions for specific SNPs/indels between original tumors and MEN-Os. P#, patient sample (original tumor); PO#, patient organoid (MEN-O).

We then evaluated the transcriptomic mutations as a representation of the underlying genomic alterations. A comparative analysis at the SNP and indel levels revealed that transcriptomic mutations exhibited a high degree of similarity between the original tumors and corresponding MEN-Os (Figure 5a). Additionally, a comprehensive analysis of mutations in key meningioma-related transcripts revealed a strong concordance in mutant transcript fractions (Figure 5b), validating MEN-Os as a reliable ex-vivo platform for faithfully capturing the mutational landscape of meningiomas.

## 4. Discussion

Patient-derived ex vivo meningioma models are crucial for advancing our understanding of tumor pathophysiology and developing personalized therapeutic strategies. In this study, we successfully established a reproducible and highly efficient protocol for generating patient-derived MEN-Os. These MEN-Os retained a diverse population of cell types, including tumor and stromal cells, faithfully preserving the complexity of the tumor microenvironment, particularly the immune microenvironment. We demonstrated that MEN-Os maintained their biological and structural integrity over long-term culture. Histological and IHC analyses confirmed that MEN-Os closely resembled their parental tumors, preserving key architectural and molecular features. Additionally, transcriptomic profiling revealed a high degree of similarity between MEN-Os and the original tumors, including the expression of genes critical to meningioma pathology.

Meningioma pathophysiology is characterized by diverse molecular, cellular, and microenvironmental features that contribute to tumor growth, progression, and recurrence. Historically, meningiomas were classified and studied based primarily on histological characterization, which categorized tumors into three grades with limited prognostic and therapeutic implications [2]. However, recent advances in genomic and transcriptomic knowledge have shifted the focus to molecular characterization, offering deeper insights into tumor biology. Key genetic drivers, including mutations in NF2, SMO, KLF4, TRAF7, and chromosomal aberrations, allow for the stratification of meningiomas into distinct molecular subtypes, each associated with unique clinical outcomes and therapeutic vulnerabilities [4,30,33,34,35,36]. The impact of this shift has been transformative in the development of targeted therapies. For example, mutations in the SMO gene have opened avenues for using SMO inhibitors, while alterations in the PI3K/AKT/mTOR pathway have highlighted potential targets for pathway-specific drugs [37]. Despite these advances, translating molecular discoveries into effective clinical treatments remains challenging due to the lack of physiologically relevant models that replicate the complex tumor microenvironment.

Tumor organoid models have become indispensable for bridging the gap between preclinical results and clinical translatability. Unlike monoclonal cell cultures, which lack the complexity of the tumor microenvironment, or murine models, which often fail to fully replicate human tumor biology, organoids faithfully recreate the three-dimensional structure, cellular diversity, and molecular characteristics of the original tumors [18]. Crucially, organoids preserve the tumor-immune microenvironment, allowing for detailed studies of tumor-stroma and tumor-immune interactions [17,18]. Previous efforts to establish MEN-Os have faced significant methodological and reproducibility challenges. Most models relied on enzymatic dissociation of patient tissues into single-cell suspensions, which disrupted the tumor microenvironment and compromised the original cellular composition and organization of the parental tissues [21,22]. Earlier models frequently utilized medium supplemented with serum and growth factors or used Matrigel for MEN-O cultures [20,21,22], methods known to induce cellular adaptations and unpredictable phenotypic changes [38]. Another protocol demonstrated the maintenance of tumor-stroma interactions and tumoral architecture, but its findings were constrained by a limited sample size, preventing comprehensive validation [19]. These challenges underscore the need for robust, reproducible methodologies to create MEN-Os that faithfully preserve the complexity of the native tumor microenvironment.

Here, patient-resected tumor tissues were meticulously sectioned into small pieces to preserve the tumor’s native architecture and cellular diversity. This approach ensures adequate oxygenation and nutrient diffusion while minimizing tissue processing time, thereby maintaining high tissue viability. In addition to tumoral cells and stem cells, endothelial cells, likely representing the tumor’s vasculature, were preserved within the MEN-Os, alongside immune cells such as macrophages and dendritic cells. This retention of key components of the tumor microenvironment offers a unique advantage, enabling the screening of candidate drugs targeting tumor–stroma interactions, including immunotherapies [39]. The protocol’s use of non-supplemented media, devoid of serum, growth factors, or Matrigel, enhances the molecular integrity of the MEN-O by minimizing external influences on cellular behavior, allowing the model to more accurately reflect native tumor biology. Notably, the absence of these supplements resulted in slower-growing cultures compared to previously described protocols [21,22]. However, this aligns with the slow growth rate observed in the patients’ tumors, which is typically seen in human meningiomas, further validating the model’s accurate reflection of human disease [40]. Additionally, the exclusion of Matrigel—commonly employed in other organoid models—prevents undesired cellular adaptations and the preferential expansion of specific cell populations, while having no effect on organoid structural integrity and simplifying the protocol and improving its reproducibility.

MEN-Os offer several unique advantages over preclinical models, such as patient-derived xenograft (PDX) models. Unlike PDX models, which often require immunocompromised mice, MEN-Os offer the unique benefit of better preserving the native tumor microenvironment, including immune cell interactions. Additionally, MEN-Os are easier to culture and scale, providing a more versatile platform for high-throughput drug screening. While xenograft models remain valuable for studying tumor growth in vivo, MEN-Os are advantageous in their ability to provide insights into cellular heterogeneity, the tumor’s molecular characteristics, and the effects of treatments in a controlled, ex-vivo environment.

With the shift towards personalized medicine, MEN-Os hold significant potential for advancing targeted therapy. Multiple ongoing clinical trials (e.g., NCT02523014, NCT03604978, NCT04659811) are leveraging tumor-specific mutations to guide tailored drug therapies [9]. Despite these pathway mutation–drug therapy concordances, treatment efficacy does not always translate as expected. In this context, MEN-Os could serve as a valuable co-clinical model for testing patient-specific tumor responses, providing a more accurate prediction of treatment outcomes and improving the overall precision of personalized therapies.

### Limitations

This study successfully established MEN-Os from 15 patient-resected meningioma samples, which, while sufficient to demonstrate the feasibility of the model, may limit the generalizability of the findings. Expanding the cohort to include a larger sample size and a broader range of cases, such as Grade 3 meningiomas and pediatric meningiomas, could further validate the model and enhance its applicability. Although MEN-Os were maintained for up to four weeks, extending culture duration in future studies would enable the investigation of chronic treatment effects and long-term tumor behavior. Incorporating functional studies, such as drug-response assays, could also validate the utility of MEN-Os for therapeutic screening and confirm their relevance for preclinical applications. Additionally, while this study provides comprehensive bulk transcriptomic analysis, integrating single-cell sequencing and proteomic validation would offer deeper insights into MEN-O fidelity by capturing cellular heterogeneity and interactions, and elucidating protein-level functionality.

## 5. Conclusions

Overall, this work establishes a reproducible and robust platform to study meningioma biology. By preserving the structural complexity, cellular diversity, and immune components of the tumor microenvironment, MEN-Os provide a faithful representation of parental tumors. The exclusion of external confounding factors ensures the molecular integrity and reproducibility of the model. This approach not only facilitates a deeper understanding of meningioma pathophysiology but also offers a valuable tool for screening therapeutic agents, particularly those targeting tumor–stroma and tumor–immune interactions. Ultimately, MEN-Os represent a critical advancement in meningioma research, bridging the gap between preclinical studies and clinical applications, with significant potential for personalized medicine.

## Figures and Tables

**Table 1:** Patient cohort.

Sample	Age	Gender	Anatomical Location	Laterality	WHO Grade	Culture Success
P1	48	F	Sphenoid wing	Left	1	No
P2	47	M	Intraventricular	Left	1	Yes
P3	49	F	Parietal parasagittal	Left	1	Yes
P4	66	F	Parietal parasagittal	Left	2	Yes
P5A	43	M	Planum sphenoidale	Midline	1	Yes
P5B			Parietal convexity	Left	2	Yes
P6	67	M	Frontotemporal convexity	Left	1	Yes
P7	40	M	Frontal parasagittal	Right	2	Yes
P8	61	F	Planum sphenoidale	Midline	1	Yes
P9	46	M	Spinal	Midline	1	Yes
P10A	43	F	Sphenoid wing	Left	1	Yes
P10B			Olfactory groove	Midline	1	Yes
P11	54	F	Parietal convexity	Right	1	Yes
P12	47	F	Parietal parasagittal	Right	1	Yes
P13	62	M	Planum sphenoidale	Midline	1	Yes
P14	49	F	Frontal parasagittal	Left	2	Yes
P15	60	M	Interventricular	Right	1	No
P16	79	F	Frontal convexity	Right	1	No
P17	50	F	Frontoparietal convexity	Left	2	No

M, male; F, female.

## Data Availability

RNA-sequencing data is available at GSE287174.

## Supplementary Materials

The following supporting information can be downloaded at: https://www.mdpi.com/article/10.3390/cancers17030526/s1, Table S1. Reagents and antibodies. Figure S1. Initial quality-control step for high quality meningioma organoid establishment. The tumor tissue was randomly sampled followed by flash-freezing, H&E staining and histopathological examination. Tissues with high density of tumor cells were selected for establishing MEN-O, in contrary to the tumor pieces with high degree of fibers tissue which led to culture failure. Middle-panel showing tissue staining immediately after tumor resection. Right-panel showing organoids at 4 weeks. Magnification: middle-panel, 20×; right-panel, 4×. Figure S2. Gene expression correlation between original tumor tissue and corresponding established organoids. Pearson correlation between sample pairs and reported coefficients of determination R2 (*p* < 0.001). Figure S3. Meningioma cell clusters reference matrix. Cell clusters RNA-sequencing reference matrix constructed from single cell sequencing data.
