# Supplementary material for: Patient-Derived Meningioma Organoids: A Reliable Model for Studying Human Tumor Pathophysiology
Source: Cancers (Basel). 2025 Feb 5;17(3):526. doi: 10.3390/cancers17030526 (PMC11817449; doi:10.3390/cancers17030526)
Supplement: Supplementary file 1 [file cancers-17-00526-s001.zip › cancers-3440233-Supplementary Figures.pdf]

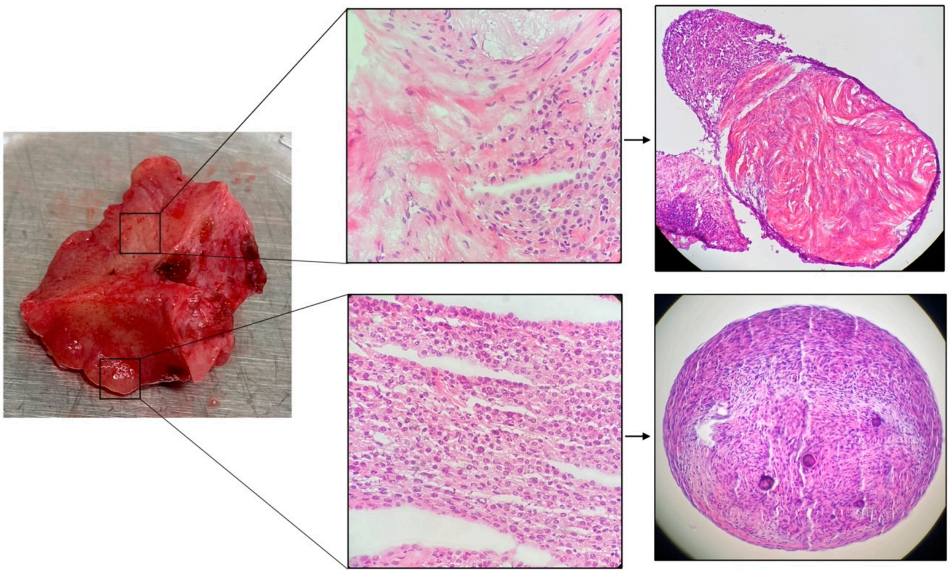

Supplementary Figure S1. Initial quality-control step for high quality meningioma organoid establishment. The tumor tissue was randomly sampled followed by flash-freezing, H&E staining and histopathological examination. Tissues with high density of tumor cells were selected for establishing MEN-O, in contrary to the tumor pieces with high degree of fibers tissue which led to culture failure. Middle-panel showing tissue staining immediately after tumor resection. Right-panel showing organoids at 4 weeks. Magnification: middle-panel, 20x; right-panel, 4x.

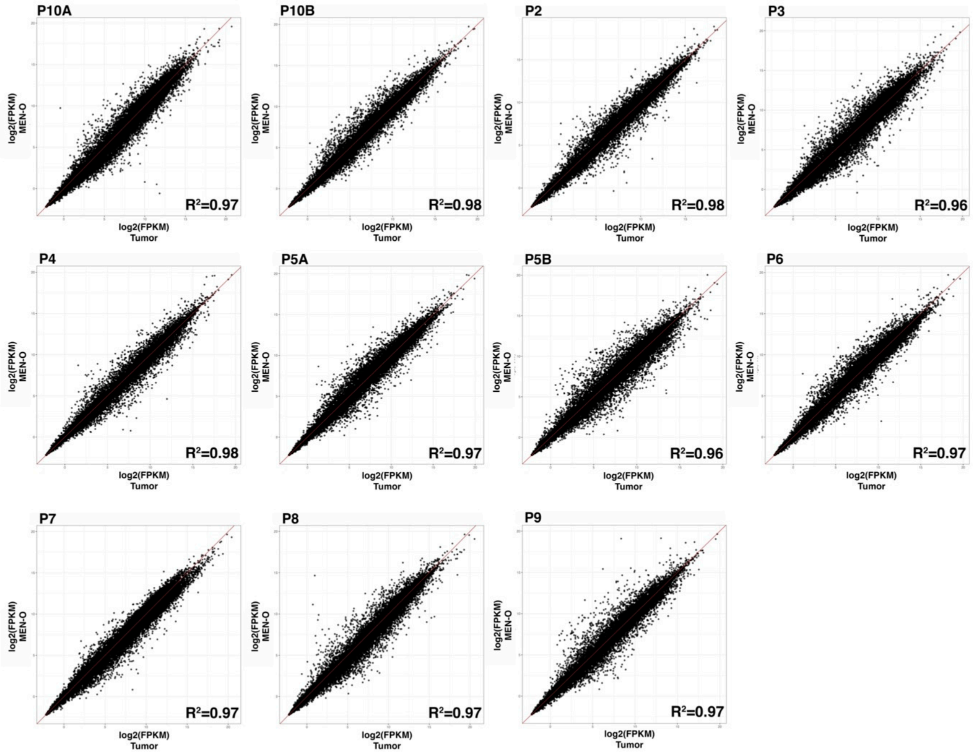

Supplementary Figure S2. Gene expression correlation between original tumor tissue and corresponding established organoids. Pearson correlation between sample pairs and reported coefficients of determination R^2 ($P<0.001$).

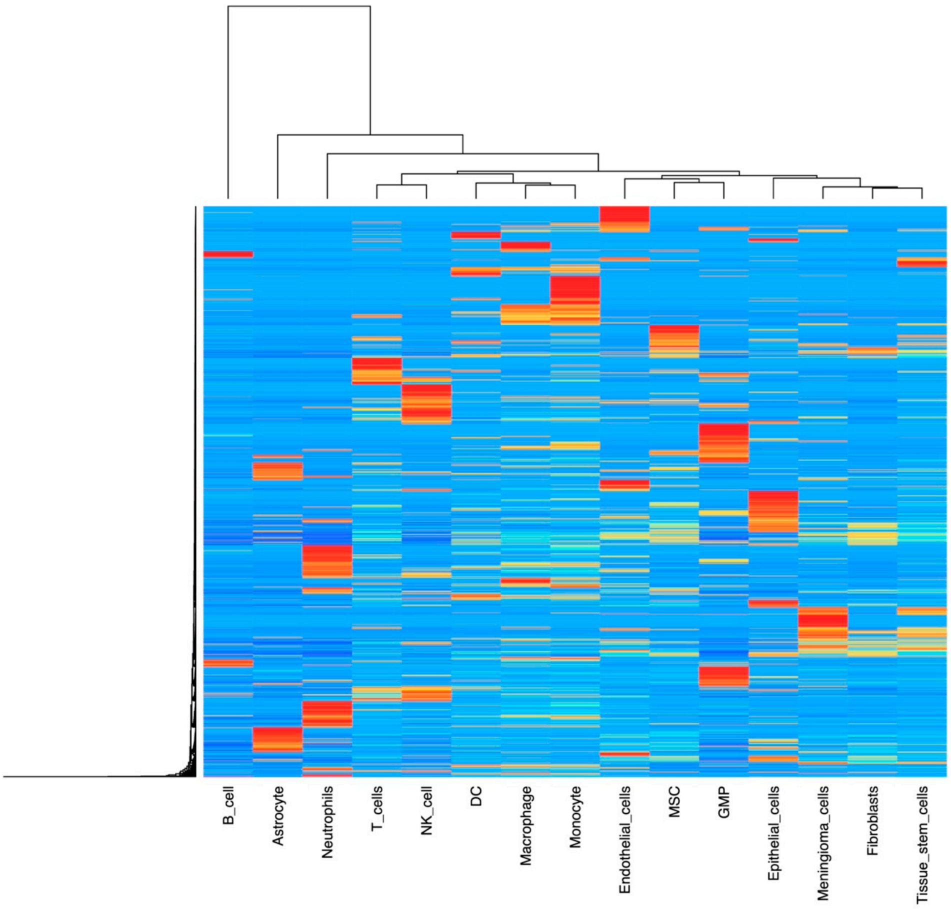

Supplementary Figure S3. Meningioma cell clusters reference matrix. Cell clusters RNA-sequencing reference matrix constructed from single cell sequencing data.
